# Differences in the microbiome of the small intestine of Leghorn lines divergently selected for antibody titer to sheep erythrocytes suggest roles for commensals in host humoral response

**DOI:** 10.3389/fphys.2023.1304051

**Published:** 2024-01-08

**Authors:** Shelly J. Nolin, Paul B. Siegel, Christopher M. Ashwell

**Affiliations:** ^1^ Prestage Department of Poultry Science, North Carolina State University, Raleigh, NC, United States; ^2^ School of Animal Science, Virginia Polytechnic Institute and State University, Blacksburg, VA, United States; ^3^ Davis College of Agriculture, Natural Resources, and Design, West Virginia University, Morgantown, WV, United States

**Keywords:** microbiome, antibody response, VA tech HAS and LAS, machine learning, immunogenetics

## Abstract

For forty generations, two lines of White Leghorn chickens have been selected for high (HAS) or low (LAS) antibody response to a low dose injection of sheep red blood cells (SRBCs). Their gut is home to billons of microorganisms and the largest number of immune cells in the body; therefore, the objective of this experiment was to gain understanding of the ways the microbiome may influence the differential antibody response observed in these lines. We achieved this by characterizing the small intestinal microbiome of HAS and LAS chickens, determining their functional microbiome profiles, and by using machine learning to identify microbes which best differentiate HAS from LAS and associating the abundance of those microbes with host gene expression. Microbiome sequencing revealed greater diversity in LAS but statistically higher abundance of several strains, particularly those of *Lactobacillus,* in HAS. Enrichment of microbial metabolites implicated in immune response such as lactic acid, short chain fatty acids, amino acids, and vitamins were different between HAS and LAS. The abundance of several microbial strains corresponds to enriched host gene expression pathways related to immune response. These data provide a compelling argument that the microbiome is both likely affected by host divergent genetic selection and that it exerts influence on host antibody response by various mechanisms.

## Introduction

Gut microbes are integral to intestinal physiology. Necessary for maintaining homeostatic balance between roles of nutrient absorption and pathogen response; resident microbes train the intestine to tolerate commensals while recognizing and responding to pathogens (Shulzhenko et al., 2011; Oakley et al., 2014). The accessibility of next-generation tools, such as 16S ribosomal RNA sequencing, has facilitated better characterization of chicken microbiomes and correlated associations between commensal microbes and bird health and physiology (Benson et al., 2010; Oakley et al., 2014; Al-Marzooqi et al., 2020). Research is emerging which further connects host humoral response to vaccines and the commensal microbiota (Zimmermann and Curtis, 2018; Gonçalves et al., 2021; Jordan, Carding, and Hall, 2022).

Lines of White Leghorns have been divergently selected for high or low antibody response 5 days post injection with intravenous sheep red blood cells (Siegel and Gross, 1980). These lines, HAS and LAS, have been under constant divergent selection for over 40 generations and are valuable models for avian immunology and genetics, however the majority of the phenotypic differences remain unexplained (Gehad et al., 1999; Dorshorst, Siegel, and Ashwell, 2011; Lillie et al., 2017). Fecal 16S sequencing of HAS and LAS, along with associated lines where selection was relaxed, HAR and LAR, show selection associated differences in microbial abundance (L. Yang et al., 2017). Understanding how selection affects changes in microbiome composition and abundance is important in exploring the host microbe relationship. Equally important however, is unravelling ways in which the resident microbes may influence host phenotype.

In addition to simply characterizing the resident microbes, there is much interest in functional analysis to better explain host-microbe interactions. For amplicon-based sequencing, functional analysis is inferentially determined using software tools such as Phylogenetic Investigation of Communities by Reconstruction of Unobserved States (PICRUSt) (Douglas, Beiko, and Langille, 2018). Functional profile inference provides more information to better understand what molecules may be involved in host-microbe interactions, however this approach has obvious limitations both in terms of incomplete information due to dependence on previously sequenced microbial genomes as well as understanding how microbial gene products may affect host physiology. More integrative approaches are necessary to make accurate associations between microbes and host molecular responses.

Machine learning is revolutionizing omics data analysis (Lin and Lane, 2017; Leite et al., 2018; Oh et al., 2021). Predictive algorithms make it possible to identify patterns in large data sets and make relevant associations. This is useful in microbiome data analysis because the abundance of specific microbes can identify microbial “signatures” specific to, and able to differentiate between, two or more groups. This analysis can complement conventional methods to define which microbes best characterize a phenotypic group. Additionally, once microbial signatures are identified, that information can be used as the “classifier” for subsequent machine learning with other data. We have RNA sequencing data for the jejuna segments from which the microbes for 16S sequencing were obtained (Nolin Shelly et al., 2023). Machine learning utilizing microbial signatures with gene expression data, enables the identification of patterns of gene expression which best predict microbe abundance. By determining what molecular pathways are altered by genes differentially expressed in chickens with specific microbial signatures we are then able to make associations of host gene expression with commensal microbiomes. The integration of microbial data, with genetic line and gene expression data becomes a novel means to further explain how microbes may influence underlying host physiology and in turn host phenotype.

## Materials and methods

### Animal work

Eggs from the 40th generations of lines HAS and LAS were obtained from Virginia Polytechnic Institute and State University and co-incubated until hatch. At hatch, all chicks were tagged for line identification and randomly transferred to battery cages such that chickens from both lines were raised together for the duration of the experiment. At 46 days of age, six chickens from each line were randomly selected, euthanized, and intestinal content samples collected from the duodenum, jejunum, and ileum for microbial DNA isolation. Corresponding intestinal tissue samples were collected for RNA isolation. This time point was selected because it is the traditional age of selection and the age corresponding to that of a cohort of chickens which had been injected with SRBC 5 days prior. All animal research was done in accordance with the North Carolina State University Institutional Animal Care and Use Committee.

### Nucleic acid isolation, quantitation, and sequencing

Microbial DNA was isolated from intestinal contents using the QiaAmp DNA Stool Mini Kit and RNA was isolated from each jejunum sample using the RNEasy mini kit (Qiagen, Hilden, Germany) using the manufacturer’s protocols. RNA and microbial DNA quantity were measured, and purity was assessed using the NanoDrop ND2000 spectrophotometer (Thermo Fisher Scientific, Waltham MA). Five hundred nanograms of each sample for 16S rRNA sequencing targeting the V3-V4 hypervariable region were sent to the University of North Carolina Microbiome center for library preparation, barcoding, and pyrosequencing on the Roche 454 sequencer. Two micrograms of RNA from each sample were taken to the North Carolina State University Genomics Sciences Laboratory for library preparation and sequencing on the Illumina HiSeq 2,500 sequencer.

### Sequencing data analysis

CLC Genomics Workbench (Qiagen, Hilden, Germany) was used for all sequencing and statistical analyses. Statistical comparisons use a generalized linear model and calculate a *p*-value as well as false discovery rate *p*-value (FDRp) and Bonferroni correction to correct for multiple testing.

16S amplicon sequencing data was analyzed using the CLC Microbial Genomics Module. Sequence reads passing quality control were grouped into operational taxonomic units (OTUs) based on 97% sequence similarity to reference database SILVA SSU 99 version 138.1 (Pruesse et al., 2007; Quast et al., 2012; Yilmaz et al., 2014; Glöckner et al., 2017) and representative sequences were selected from each OTU for taxonomic assignment. Metrics were calculated for alpha (within line) diversity using total number of OTUs and beta (between line) diversity using weighted unifrac. Differences in OTU abundance between HAS and LAS were considered significant at *p* ≤ 0.05. Additionally, weighted unifrac permutational multivariate analysis of variance (PERMANOVA) was run to investigate line by tissue differences and were also considered significant at *p* ≤ 0.05.

The microbial genomics module also contains a tool for inferring functional microbial profiles utilizing PICRUSt2 (Douglas et al., 2020). By importing a table containing Kmer frequency profiles with term multipliers and associated 16S copy numbers, the software can produce approximate functional profiles for mapped OTUs. This tool was used to create functional profiles as represented by KEGG (Kyoto encyclopedia of genes and genomes) (M. Kanehisa and Goto, 2000; Kanehisa, 2019; Kanehisa et al., 2021) identifiers present for HAS and LAS. Differential abundance of microbial molecules was calculated using the same GLM tool for determining OTU abundance. Due to the large number of functional molecules as well as the inferential nature of the data, the most conservative cut-off, Bonferroni correction ≤0.05 was considered significant. Differentially abundant KEGG molecules were entered into the KEGG orthology database and analyzed for module enrichment between lines.

CLC Genomics Workbench version 11 (Qiagen, Hilden, Germany) was used for RNA sequencing analysis as described previously (Nolin Shelly et al., 2023). High quality RNA sequencing reads were mapped to the Galgal6 reference genome (GCA_000002315.5) and differentially expressed genes were determined between lines, with FDRp ≤0.05 considered statistically significant.

### Machine learning

Waikato Environment for Knowledge Analysis suite (WEKA) (Hall et al., 2009) was used for Machine learning. Three algorithms were used: support vector machines, artificial neural networks, and a decision tree. Algorithms were validated using two cross validation methods, a %-split and a K-fold stratified hold-out. A % split cross validation randomly splits the data into training or test. In our experiment we used a 66%–34% split, wherein 66% of the data was assigned to be the training set and the remaining 34% used to test. Sample groups are equally represented in the test and training sets. The K-fold stratified hold-out works by randomly dividing the data into K datasets, where K-1 datasets are used for training with the remaining 1 “hold-out” dataset as the test set. For our experiment, 6 is the number of biological replicates for each line/tissue, so K = 6. This algorithm runs sequentially 6 times, such that each dataset is the test set only once and included in the training set the remainder; performance is given as the average for the 6 runs. Performance for all machine learning is calculated as the average for the three algorithms and two validation methods.

To utilize machine learning to associate microbes with genetic line and later host gene expression, first we ran the algorithms to determine which jejunal microbes were most predictive of line. We started by running the algorithms with all OTUs to determine the predictive performance using the entire dataset. Next the OTUs were ranked by combining the entropy based InfoGainAttribute ranker function in WEKA (Li et al., 2004) with the *p*-value which was calculated for differential abundance for each OTU. A portion of OTUs were then removed (those with the highest *p*-value and lowest entropy) and the algorithms were re-run with the smaller set of OTUs. This process was repeated until the optimal microbial signatures were determined for highest prediction performance. This is known as reduction of data dimensionality and allows for identifying the optimal microbial “signature” for line prediction. Once these microbes were identified, they were each used as the sole attribute for running the algorithms to determine their individual predictive performance. Microbes with <75% correct prediction performance were eliminated from further analysis, as were those exclusively present in only one line.

These remaining OTUs were divided into groups by their relative abundance as high, low, or absent, or if the microbe was present in all samples as high, medium or low, as shown in Table 1. Abundance could then be used as the classifier and each sample, previously identified as HAS# or LAS#, was now identified by relative OTU abundance. OTU counts greater than the mean (for all present samples) were classified as high and those less than the mean as low, samples which did not contain any OTU counts were obviously grouped as absent. For microbes which were present in all samples, high was calculated as the mean OTU counts +50%, low as mean −50%, and medium were counts in between. Dividing the abundances for the microbes into three groups was necessary to avoid samples being classified solely by line. RNA sequence data for each sample was then used as the attributes with microbial abundance as the classifiers for machine learning in order to determine the gene expression patterns which predict microbe abundance. As statistical analysis of differential gene expression could not be performed based on microbial abundance, the InfoGainAttribute ranker function in WEKA (Li et al., 2004) was used to rank the genes for purposes of reducing data dimensionality as before. The optimal gene lists for each microbe were run in Ingenuity Pathway Analysis (IPA, Qiagen, Hilden, Germany) to determine enriched host pathways.

**TABLE 1:** Explanation of how samples were identified by microbial abundance as, absent (A), low (L), medium (M), or high (H).

OTU	HAS1	HAS2	HAS3	HAS4	HAS5	HAS6	LAS1	LAS2	LAS3	LAS4	LAS5	LAS6
1	A	L	A	A	A	A	H	H	H	L	L	H
2	M	H	H	H	M	M	L	L	L	L	L	L
3	M	H	H	H	M	M	M	L	L	L	L	L
4	M	H	H	H	M	M	M	M	L	L	M	M
5	L	A	A	A	L	L	H	H	L	A	L	L
6	L	H	H	H	H	M	M	L	L	L	M	L
7	L	H	L	H	H	L	L	A	L	A	L	L
8	H	L	H	H	H	L	A	L	L	A	A	L
9	L	L	H	H	H	A	A	A	L	L	L	A

Negative controls for machine learning were also run for line prediction and microbial abundance. Datasets were randomized, wherein each sample identifier was randomly assigned to the data for a different sample, this allows for disassociation between classifier and attributes. Ten random datasets were used for each comparison and the average performance was calculated for each of the ten runs. Given two groups, HAS *versus* LAS 50% correct prediction would indicate random chance, whereas for microbial abundance with three classifiers, it would be ∼33%. If the negative controls performance is close to that of random probability, we can be confident that true machine learning occurred in our experimental data sets.

## Results

### Microbial diversity and abundance

**FIGURE 1:**
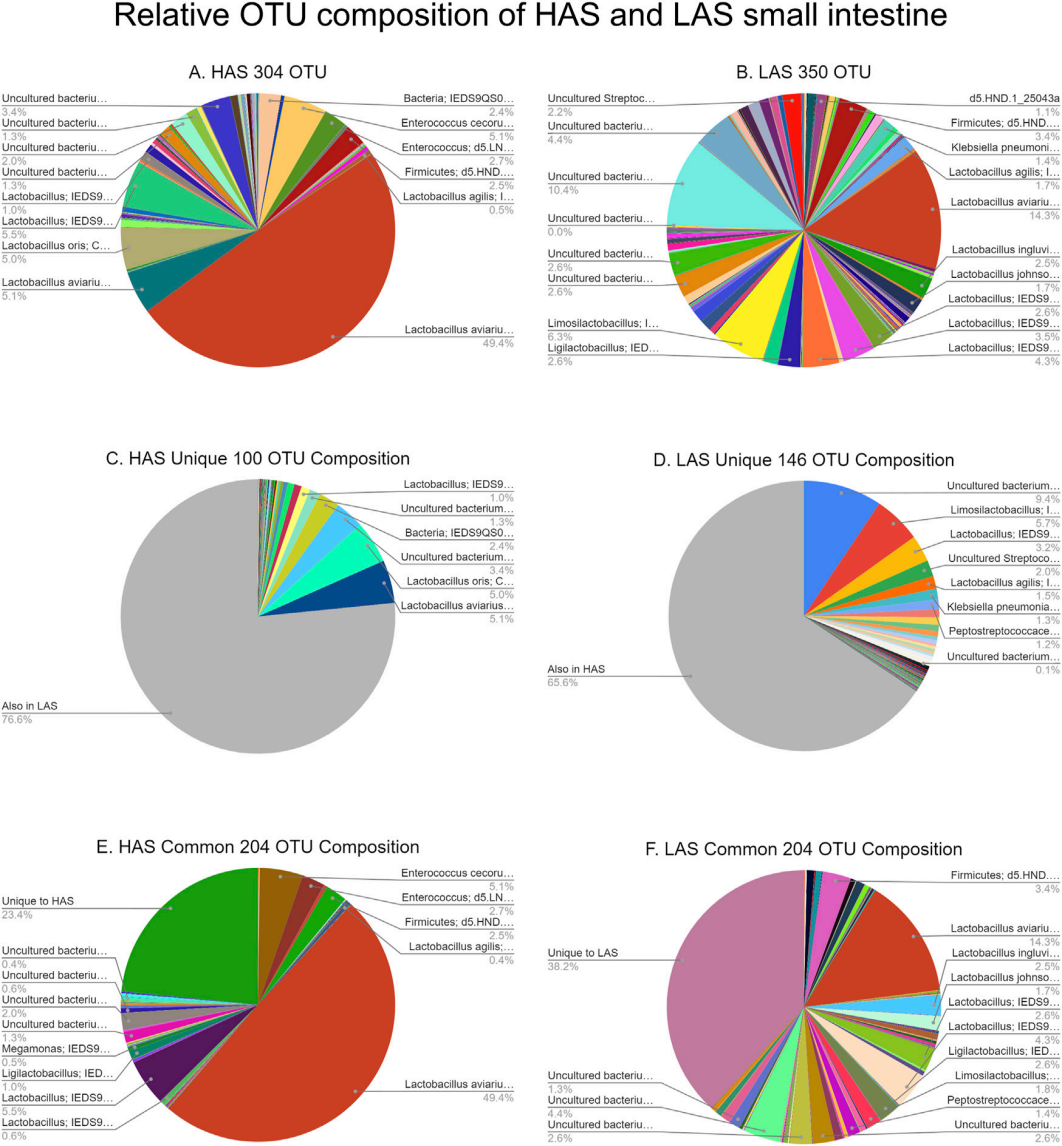
**(A–F)** Relative OTU composition of HAS and LAS small intestine.

**FIGURE 2:**
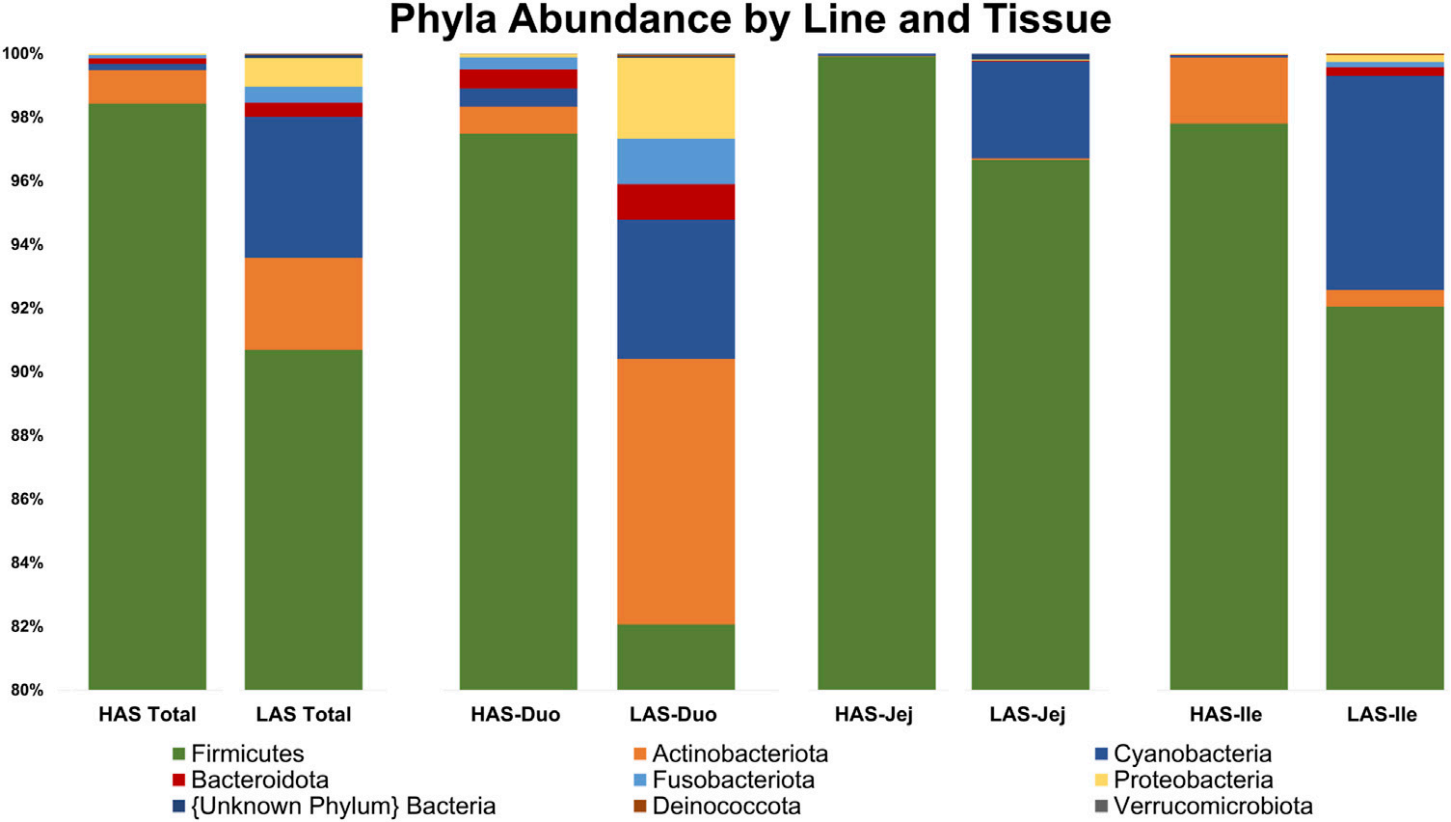
Relative abundance of the top 20% of microbes at the Phylum level in HAS and LAS total and by intestinal segment.

**FIGURE 3:**
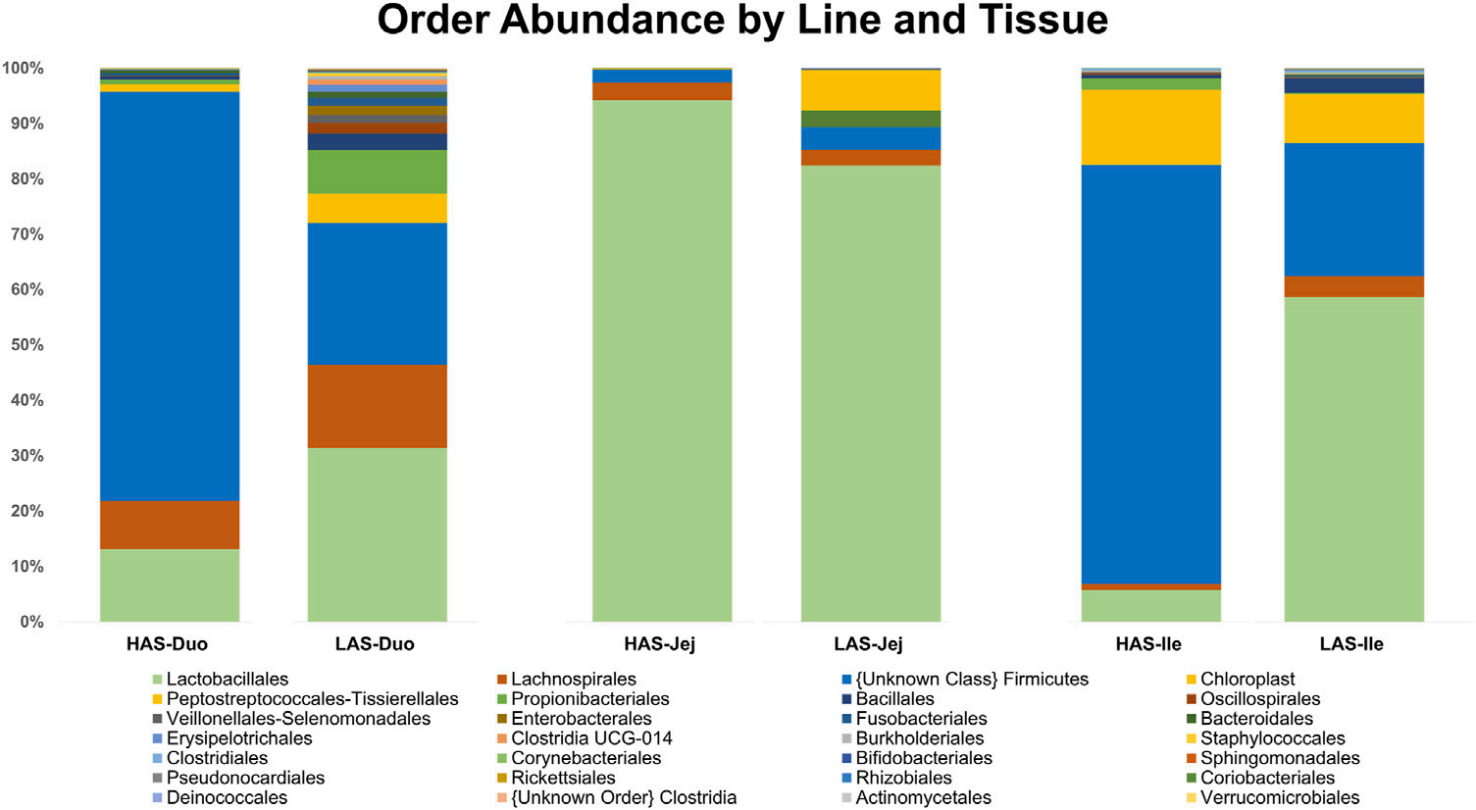
Relative abundance of microbes at the Order level in HAS and LAS by intestinal segment.

A total of 2,543 OTUs were identified, however those with less than 10 total OTU counts were filtered out, leaving 450 OTUs remaining for further analysis. LAS had the slightly more diverse microbiota of the two lines, with 350 OTUs present *versus* 304 OTUs present in HAS. There was overlap of 204 OTUs common to both lines, leaving a substantial percentage of the microbiota unique to one line or the other, see Figures 1A–F. As previously reported (Yang et al., 2017), *firmicutes* were the most abundant phyla in both lines, as well as for all intestinal segments, accounting for >80% of all microbes in our samples, followed by *actinobacteria*, *cyanobacteria*, and *proteobacteria* as shown in Figure 2. Within the *firmicutes*, lactic acid bacteria of the closely related genera *Lactobacillus*, *Ligilactobacillus*, and *Limosilactobacillus* are by far the most dominant for HAS and LAS, both in terms of relative abundance (>95%) as well as number of individual OTUs. Each intestinal segment also had a microbial signature unique from the others. At the order level, the duodenum had the most diversity, followed by the ileum, and very little diversity was found in the jejunum. These data are shown in Figure 3.

**FIGURE 4:**
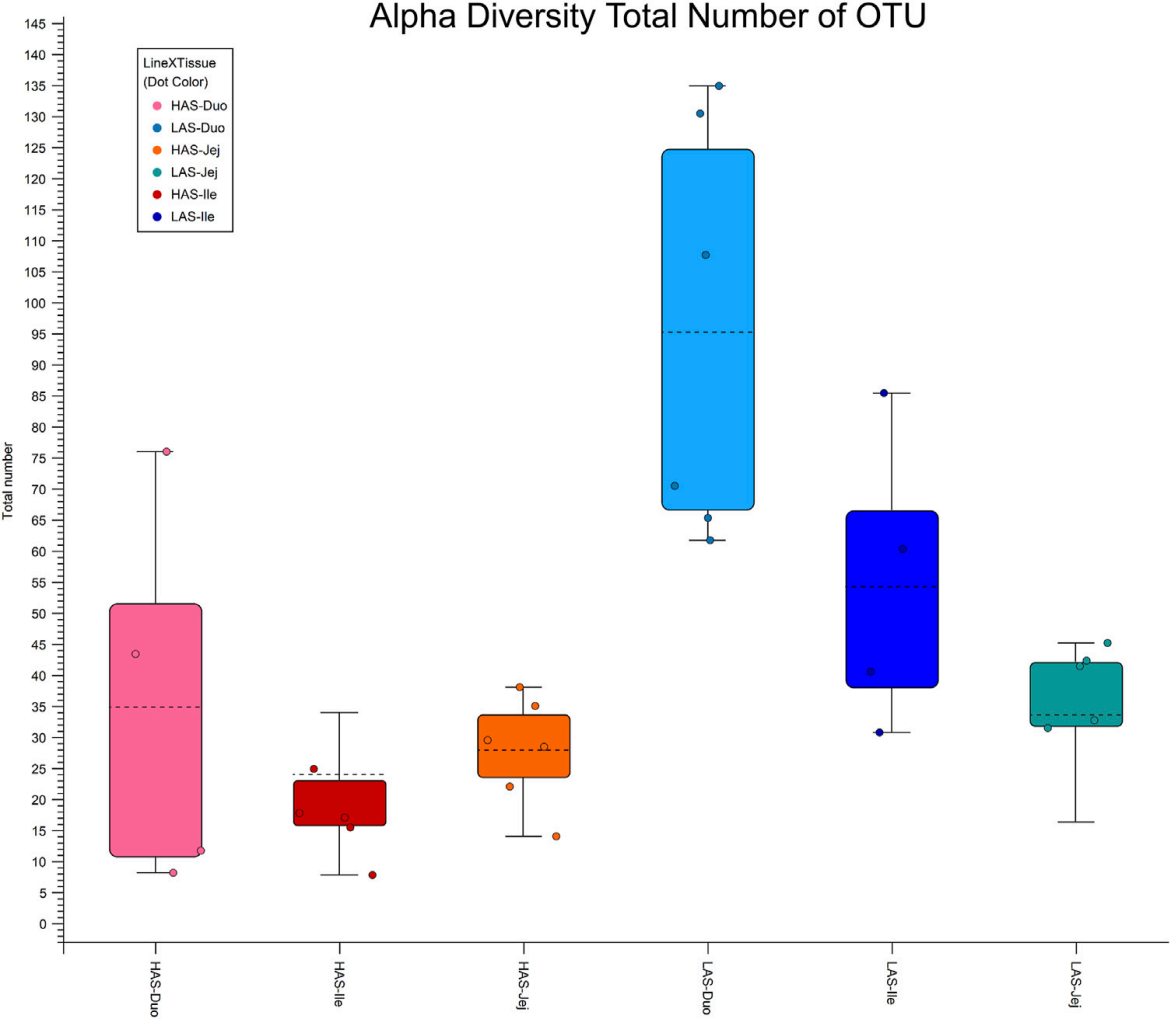
Alpha diversity plot total number of OTUs for duodenum, jejunum and ileum in lines HAS and LAS.

Alpha diversity box plots in Figure 4 further illustrate the microbial richness of LAS vs. HAS, as the total number of OTUs observed for LAS samples are greater than those of HAS for each intestinal segment. Additionally, while rarefaction curves are not shown, the diversity captured in HAS seems thorough, whereas additional sequencing in LAS may have identified additional OTUs.

**FIGURE 5:**
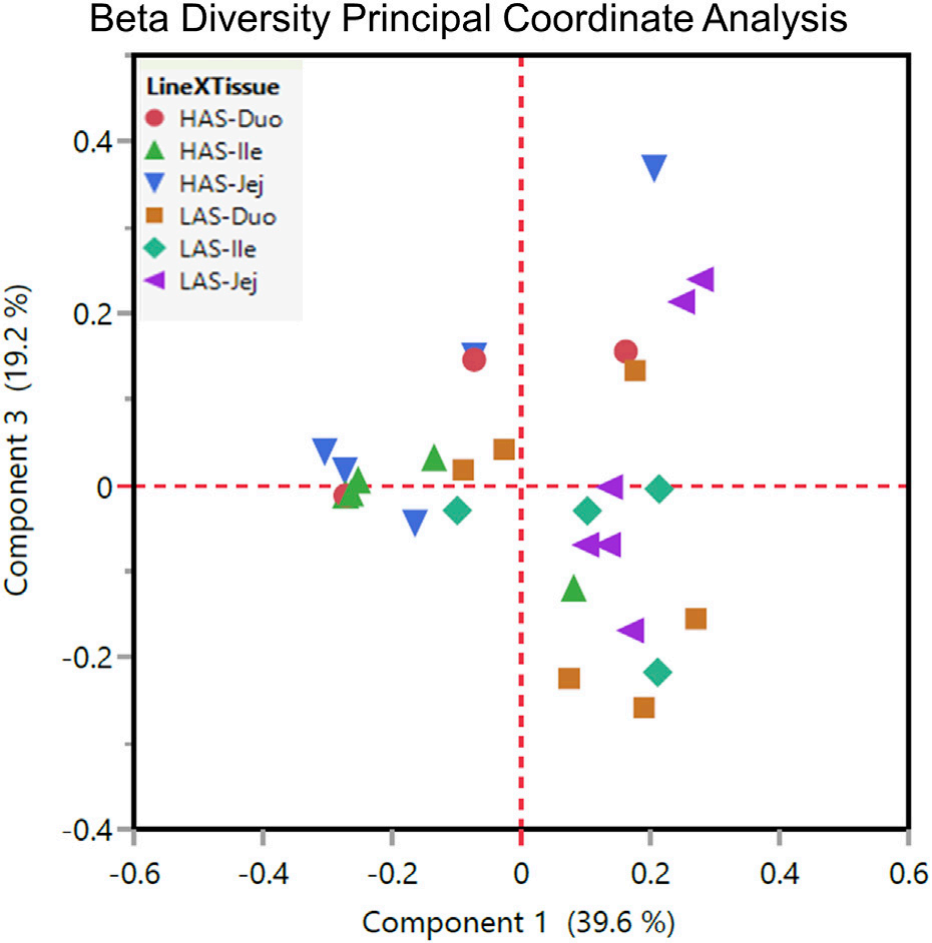
Beta diversity plot principal coordinate analysis for all HAS and LAS duodenum, jejunum, and ileum sample.

Beta diversity, variation between samples, as shown by principal coordinate analysis in Figure 5 illustrates how the samples cluster by tissue and line. In general, it seems that within line microbial differences became less prominent while between line differences became more prominent from anterior to distal segments, given that samples cluster somewhat more closely in the ileum and jejunum than in the duodenum, particularly so for the HAS line.

### Statistical differences in abundance

**FIGURE 6:**
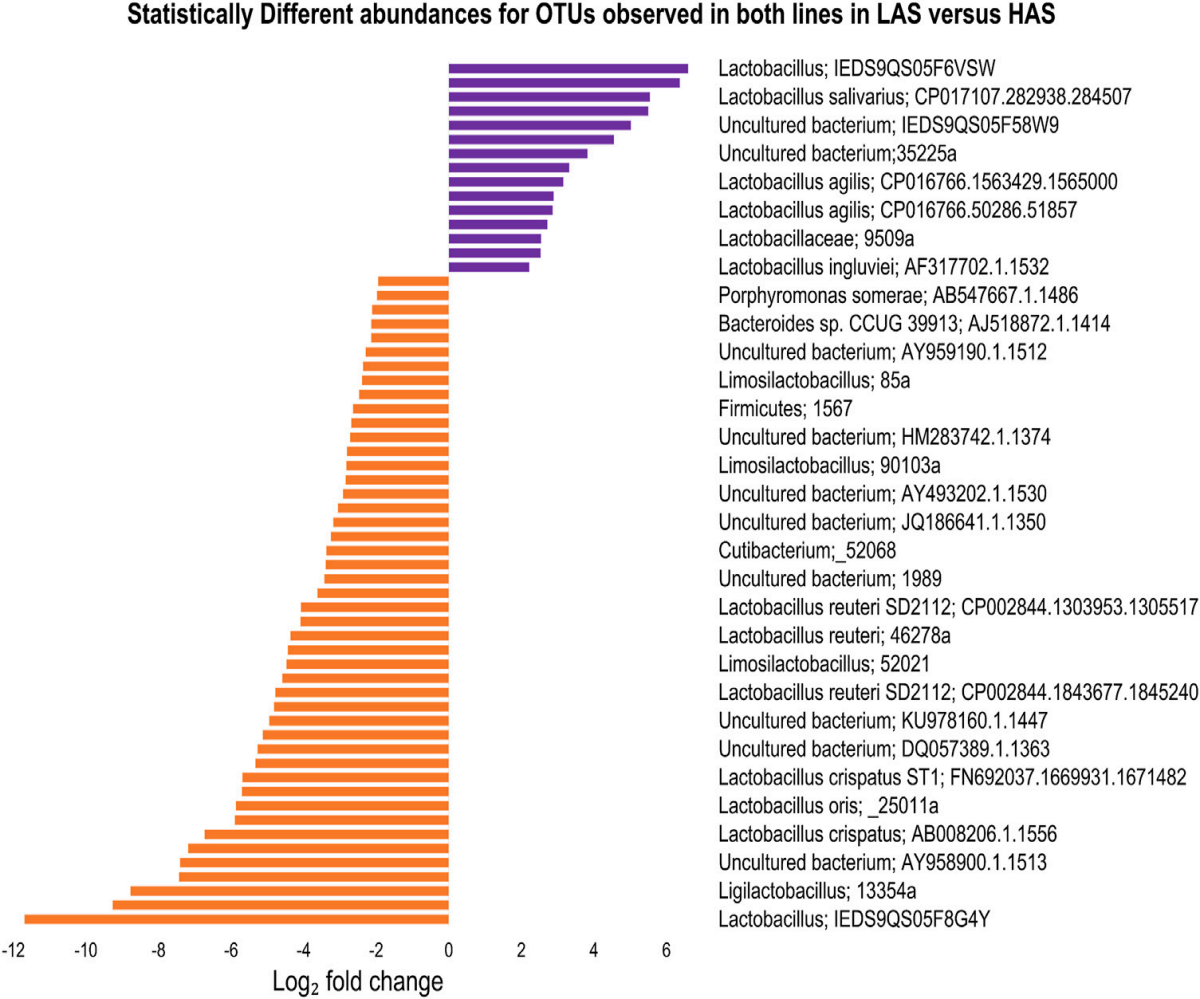
Statistically different microbes between HAS and LAS for OTUs observed in both lines in any intestinal segment.

We found 206 OTUs which differed in abundance between lines, irrespective of tissue, at FDRp ≤0.05. Of those, 65 OTUs were observed in both lines. In contrast to most of the data where LAS had exhibited more diversity only 18 of the common OTUs were more abundant in LAS while 47 were more abundant in HAS as shown in Figure 6. Additionally, 100 of the 206 OTUs only present in one line were found exclusively in HAS while 41 were only found in LAS.

PERMANOVA revealed statistically significant line by tissue differences. The jejunum and ileum were significantly different between lines, though there was not a significant difference between the duodenum of HAS *versus* LAS. Within line, significant differences were observed between HAS duodenum and jejunum, and between LAS jejunum and ileum.

### Functional analysis

Functional analysis identified 1385 KEGG molecules which were differentially abundant between lines; 704 more abundant in LAS *versus* 681 more abundant in HAS. Using the KEGG mapper tool for each list of molecules revealed enrichment of modules for different types of metabolic pathways between lines. Of the top 10 modules in HAS five were associated with biosynthesis of molecules fatty acid, lysine, pyrimidine, riboflavin, and coenzyme A. Four were involved in the 3-Hydroxypropionate bi-cycle, glycolysis, antimicrobial resistance, and ATPase activity. Conversely, LAS had three modules involved in degradation: AMP, GMP, and phenylacetate. Three modules were enriched for reductive carbon conversion, two were involved in menaquinone biosynthesis, and one in NAD biosynthesis. LAS and HAS both had enriched modules for C5 isoprenoid, though HAS was enriched for the mevalonate pathway whereas the non-mevalonate pathway was enriched in LAS.

### Machine learning and host pathway analysis

**FIGURE 7:**
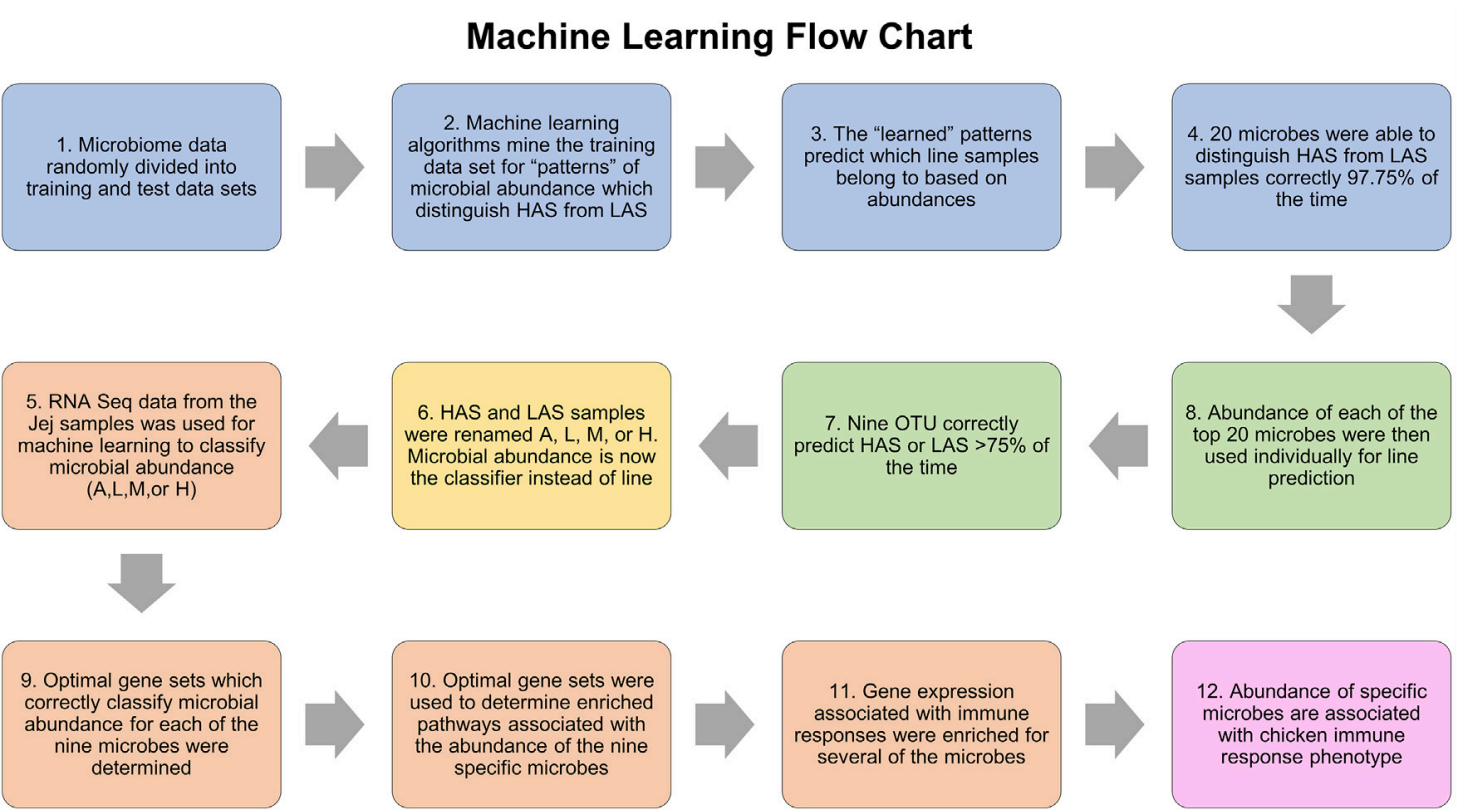
Flowchart of the machine learning process.

Figure 7 summarizes the machine learning workflow. In the jejunum, the combination of the top twenty OTUs identified via machine learning resulted in an average predictive performance of 98.61%, that is to say the algorithms were able to identify patterns in the abundance data for these microbes which could be used to accurately classify the samples as being from the HAS line or LAS line on average 98.61% of the time. The randomized datasets performed at an average of 45%, which is very close to the 50% expected by random probability and assured us that “true” machine learning occurred. Of those top twenty OTUs, eleven had average individual predictive performance >75%, however two were only present in the LAS line. The nine remaining OTUs which were both predictive of line and present in at least one sample from each line included eight uncultured strains of the genus *Lactobacillus* identified as EU776306.1.1423, AY958858.1.1540, EU460910.1.1421, EU774852.1.1423, EU774852.1.1423, IEDS9QS05FX4Q0, sp. HM218868.1.1554, and HM218952.1.1524, one species of *Lactobacillus crispatus*: EU559595.1.1562, and one of the genus *Enterococcus* EU459393.1.1420 (also uncultured). These OTUs were subsequently used to identify host gene expression associated with the relative abundance of each microbe, which were used as input for pathway enrichment analysis.

Each OTU dataset was run independently using the three machine learning algorithms and two cross validation methods. The relative abundance of microbes was used as the classifier (absent, low, medium, or high) and the predictive dataset attributes were composed of the gene expression data in transcripts per million. RNA was sequenced from the jejunum tissue samples, the contents from which the microbial DNA was isolated. The list of genes whose expression patterns most accurately predicted relative OTU abundance was determined for each of the nine OTUs and used for pathway enrichment analysis. Predictive performance for each optimized gene list was between 67% and 90%. Genes lists varied from 35–500 and IPA was able to map >60% of those in each list. The randomized negative control dataset performances were between 27% and 38%, close to the expected 33.3% indicative of random chance. The results of machine learning and IPA are summarized in Table 2, and all metrics for the machine learning algorithm runs with optimized feature sets can be found in Supplementary Table S1.

**TABLE 2:** Functional pathway enrichment for genes associated with most predictive microbes.

Taxa	% Correct line prediction	Top ave % performance for RNASeq	# Genes for maximum performance	#IPA mapped genes	Top enriched pathways
Ent_UnCul_EU459393.1.1420	90	82	339	244	Neuroinflammation Signaling Pathway, Antigen Presentation Pathway, Virus Entry via Endocytic Pathways
LB_crispatus_EU559595.1.1562	83	85	35	22	Flavin Biosynthesis IV, Tetrapyrrole Biosynthesis II, Selenocysteine Biosynthesis II
LB_UnCul_EU776306.1.1423	78	78	325	253	Glycolysis I, Colanic Acid Building Blocks Biosynthesis, Heme Degradation
LB_UnCul_AY958858.1.1540	88	74	349	264	Gnaq Signaling, fMLP Signaling in Neutrophils, IL-1 Signaling
LB_UnCul_EU460910.1.1421	76	88	83	58	Synaptogenesis Signaling Pathway, Neuregulin Signaling, UDP-N-acetyl-D-galactosamine Biosynthesis II
LB_UnCul_EU774852.1.1423	79	85	500	444	Role of Macrophages, Fibroblasts and Endothelial Cells in Rheumatoid Arthritis, ERK/MAPK Signaling, Role of Osteoblasts, Osteoclasts and Chondrocytes in Rheumatoid Arthritis
LB_UnCul_IEDS9QS05FX4Q0	76	90	228	186	Phagosome Maturation, Antigen Presentation Pathway, Colanic Acid Building Blocks Biosynthesis
LB_UnCul_sp.HM218868.1.1554	79	75	312	219	WNT/B-catenin Signaling, Neuroinflammation Signaling Pathway, Stearate Biosynthesis I, (SLC25A51)
LB_UnCul_HM218952.1.1524	78	67	45	40	NADH Repair, Salvage Pathways of Pyrimidine Deoxyribonucleotides, Insulin Secretion Signaling Pathway

## Discussion

Forty generations of divergent selection have resulted in a greater than six-fold difference in 5 day post injection antibody titer to sheep red blood cells between the HAS and LAS lines. Previous research has identified differences which help explain the phenotype, including their major histocompatibility B-haplotypes, as well as other genetic loci identified via pooled resequencing and quantitative trait loci mapping of an advanced intercross line (Gehad et al., 1999; Dorshorst, Siegel, and Ashwell, 2011; Lillie et al., 2017). However, much of the variation in antibody response has yet to be elucidated. The microbiome has become a key area of immunology research, and given that commensal microbes are thought to coevolve with their host (Zhao et al., 2013; Meng et al., 2014; Yang et al., 2017; Koskella and Joy, 2020), and influence host humoral response to vaccines (Zimmermann and Curtis, 2018; Gonçalves et al., 2021) the microbiomes of HAS and LAS likely play a role in their differential antibody response phenotypes.

Sequencing the small intestinal microbiome of HAS and LAS has demonstrated an association between genetic selection and microbial composition and diversity. The microbiome of the small intestine were distinctly different between lines HAS and LAS, wherein LAS exhibited greater microbial diversity than HAS, in agreement with previous fecal microbiome research (Zhao et al., 2013). Genetic selection for high or low antibody response to SRBCs appears to have resulted in an inverse relationship with commensal microbe diversity. While LAS exhibited greater numerical diversity, it is also important to note that from a statistical standpoint HAS displayed an increased abundance of more microbial strains. The discordant observances in statistical *versus* numerical abundance may be attributed to greater within line diversity in LAS than HAS. That is to say, HAS may have more microbes which differ statistically from LAS, but that is because LAS samples differ more from one another than HAS samples do. This is further supported by the results of the alpha diversity analysis, where the diversity captured in the HAS samples appears more comprehensive.

*Lactobacillus*, being a predominant genus in both lines, warrants additional exploration. The species/strains of lactobacilli present in one line are often significantly reduced or entirely absent in the other and it is important to consider the additive effects of small differences in microbial abundance and composition that may contribute to host physiology. Studies of feeding different probiotic *Lactobacillus* strains result in various degrees and types of immunostimulant (Perdigón, Fuller, and Raya, 2001). One of the main ways microbes exert an effect on host immune response in the gut is via fermentation products such as lactic acid, and the short chain fatty acids (SCFA) (Kim, 2018). As its name suggests, *Lactobacillus* is responsible for producing lactic acid and while some strains can produce short chain fatty acids or manipulate the microbial environment leading to SCFA production by other microbes (H. Li et al., 2020) there is a negative correlation between *Lactobacillus* and SCFA content, particularly butyrate, in the small intestine of poultry (H. Yang et al., 2018; Du et al., 2020; Ranjitkar et al., 2016). Butyrate, and to a lesser degree propionate, have been reported to inhibit dendritic cells, T helper cells, and B cells (Millard et al., 2002; Singh et al., 2010; Arpaia et al., 2013; Trompette et al., 2014; Sanchez et al., 2020) all of which would contribute to diminished immunoglobulin production. If the intestinal microbiome composition of HAS favors a reduction in SCFA producing bacteria compared to LAS, it could help explain the enhanced antibody response to sheep red blood cells observed in that line. Conversely, while intestinal lactic acid concentration has not been studied specifically in regard to antibody response, there are data to indicate that it may contribute positively. Cell culture experiments with supplemented lactic acid resulted in an increase in antibody production (Kromenaker and Frirdrich, 1994). Children suffering from rotavirus induce diarrhea given oral probiotic *Lactobacillus gg* exhibited an increase in antibody secreting cells (Kaila et al., 1992) and broilers fed probiotic *Lactobacillus bulgaris* had increased antibody titer to Newcastle’s Disease Virus vaccination (Apata, 2008). Additionally lactic acid bacteria are currently being investigated as a useful vector for vaccines (Szatraj, Szczepankowska, and Chmielewska-Jeznach, 2017). Whether it is a result of the higher levels of lactic acid, or some other metabolite produced by *Lactobacillus*, the antibody response to SRBCs observed in HAS is likely influenced by the increased abundance of commensal *Lactobacillus*.

Other microbial metabolites can also stimulate host physiology. Modules for microbial riboflavin and lysine biosynthesis were more enriched in HAS compared to LAS, which have been shown to impact immune cell function and antibody response. Antibodies to S*almonella pullorum* were observed to be impaired in animals with diets deficient in riboflavin (Panda and Combs, 1963) and influenza vaccines containing riboflavin adjuvants resulted in increased antibody titers (Quintilio et al., 2016). Additionally, microbes have been shown to present riboflavin metabolites to the MHCI related protein (MR1) of mucosal associated invariant T-cells (Treiner et al., 2003; Toubal et al., 2019). Lysine deficiency has been associated with reduced antibody response to Newcastle disease in broilers, and chickens on diets with supplemented lysine were found to have an increase in antibody titer (Chen, Sander, and Dale, 2003; Faluyi et al., 2015). Future studies may include full metagenomic sequencing to better characterize the spectrum of gut microbial metabolites which may impact chicken antibody response.

Machine learning allows for the addition of host pathway analysis related to microbe abundance. These data further support the involvement of commensal microbes in antibody response as the abundance of the microbial strains most predictive of host line are associated with enriched expression of host genes involved in antigen presentation, inflammation, and immune cell responses. Future studies may look to better determine how these specific microbes may be impacting the molecular pathways identified using this integrated machine learning approach.

By taking an integrated approach which includes 16S microbiome sequencing, functional microbiome analysis, and machine learning to characterizing the small intestinal microbiome of genetically selected lines, HAS and LAS we have been able to demonstrate differences which may influence antibody response to SRBCs. In agreement with the results of fecal microbiome sequencing (Yang et al., 2017) we saw an increase in microbial diversity in LAS birds and an increase in *Lactobacillus* abundance in HAS birds. In addition to characterizing the microbiota of the separate small intestinal segments *versus* the combined microbiota of feces, another novel aspect of our experiment is that the chickens in our study had not been injected with sheep red blood cells, thus the differences we observe are native and not influenced by antigen exposure. Based on what has been published with regard to *Lactobacillus* and microbial metabolites, our results support a role for the involvement of the associated gut microbiome with the high and low antibody response to SRBCs in these divergently selected lines. Including functional microbiome data has allowed for the inferential identification of enriched microbial modules, the products of which may influence host phenotype. Finally, machine learning allows for identifying specific microbe strains whose abundance accurately predicts line (HAS or LAS) and enables associating those microbial abundances with host gene expression data to further explore the host-microbe relationship to host antibody response. This is to our knowledge the first time these different data types have been integrated together in this way via the use of machine learning algorithms and offers a novel approach to better understanding host physiology with regard to microbiota composition.

## Data Availability

The datasets presented in this study can be found in online repositories. The names of the repository/repositories and accession number(s) can be found below: https://www.ncbi.nlm.nih.gov/, GSE206804 https://www.ncbi.nlm.nih.gov/, GSE240908.
